# The multifaceted roles of microRNAs in nonalcoholic steatohepatitis: from pathogenic mechanisms to diagnostic and therapeutic opportunities

**DOI:** 10.3389/fgene.2026.1764788

**Published:** 2026-07-10

**Authors:** Zhenkun Lu, Zhijun Li, Qianqian Liu, Shihao Du

**Affiliations:** Qingdao Public Health Clinical Center, Shandong, China

**Keywords:** diagnostic biomarkers, exosomes, microRNA, non-alcoholic steatohepatitis, therapeutic targets

## Abstract

Nonalcoholic steatohepatitis (NASH), a significant advanced stage of nonalcoholic fatty liver disease (NAFLD), has become a focal point in contemporary liver disease research due to its intricate pathological characteristics and high prevalence. MicroRNAs (miRNAs), as pivotal regulators of gene expression, play pivotal roles in the pathophysiology of lipid metabolism disorders, inflammatory responses, apoptosis, and liver fibrosis during NASH progression. Despite recent preliminary insights into the functional roles of miRNAs in NASH, their specific mechanisms and clinical applications remain challenging. This systematic Frontiers in Immunology review examines miRNA expression patterns and the signaling pathways that regulate them, focusing on the diagnostic potential of circulating and exosomal miRNAs in NASH and the latest advances in miRNA-based therapeutic strategies. The objective of the present study is to integrate multi-omics and clinical research data to provide theoretical support and practical guidance for early diagnosis and precision treatment of NASH. The ultimate aim is to advance the translational application of microRNAs in clinical settings.

## 1 Introduction

Nonalcoholic steatohepatitis (NASH) is a serious, progressive type of nonalcoholic fatty liver disease (NAFLD), with liver inflammation and scarring. NAFLD and its more serious inflammatory form, NASH, have become the most common chronic liver disease in the world, which constitutes a huge public health burden. NAFLD covers the spectrum of diseases from simple hepatic steatosis to NASH, hepatic fibrosis, liver cirrhosis, and even hepatocellular carcinoma (HCC). In the most grievous instances, the illness might cause cirrhosis, and hepatocellular carcinomas may create an acute problem for the world community. The pathophysiology of NASH is complex. In addition to problems related to lipid metabolism and the inflammatory response, there are also events involving cell death and fibrosis, with different cells participating. The common diagnosis is mainly based on the liver biopsy, which has been considered an invasive and risky method. There is currently an urgent need for noninvasive, accurate biomarkers to improve clinical diagnoses and treatment (Escutia-Gutiérrez et al., 2021; Kim et al., 2021).

MicroRNAs (miRNAs) are a class of non-coding small RNAs that regulate gene expression and are involved in pathological processes such as lipid metabolism, inflammation, apoptosis, and fibrosis (Atic et al., 2023; Morishita et al., 2023; Hochreuter et al., 2022). This led to considerable interest in their potential as diagnostic and therapeutic agents. A great deal of research has shown that miRNAs play significant regulatory roles in the development and progression of NAFLD and its more serious form, NASH. Consider miR-122 and miR-34a, for instance; miR-21 also shows anomalous expression in the liver and the circulation. This expression is closely associated with fat accumulation, inflammation, and fibrosis (Hu et al., 2024; Naeimzadeh et al., 2023; Mehmood, 2024). MiR-34a inhibitors ameliorated hepatic lipid accumulation and liver function marker levels in many animal models (Zhu et al., 2023). Again, it has been proven that miRNAs have great therapeutic potential.

Circulating miRNAs have also revealed a highly accurate non-invasive biomarker for the diagnosis of NASH. Many studies have examined the detection of serum miRNAs, as well as systematic meta-analyses, which show that serum miRNA testing yields highly sensitive and accurate results for diagnosing NASH in NAFLD. MiR-34a and miR-192 have become powerful diagnostic candidates (Kim et al., 2021; Xin et al., 2020). Many miRNA assays work better together to enhance diagnostic accuracy, opening new ways to detect diseases early and assign people into appropriate risk groups (Escutia-Gutiérrez et al., 2021). At the same time, it has been shown that the detection of specific liver extracellular vesicle miRNAs can also be significantly improved for diagnosis, as they are more tissue-specific (Newman et al., 2022).

Some advances were made in understanding the molecular mechanisms of miRNAs in NASH. The roles of miRNAs in promoting or delaying the disease process by affecting lipid metabolism, inflammation-related pathway genes, and apoptotic or fibrosis-related genes have been revealed. Take miR-942-5p and miR-193b-5p, for example: they regulate lipid accumulation and inflammation via competitive endogenous RNA (ceRNA) networks that affect NASH development (Du et al., 2024; Golchin et al., 2025). Additionally, it is shown that miRNAs participate in cellular autophagy, pyroptosis, fibrosis, etc., by regulating multiple hepatic signaling pathways, such as PTEN/AKT and LPS/TLR-4/FoxO3 (Chen et al., 2024; Samy et al., 2024). Investigating miRNA regulatory networks and their relationships with long non-coding RNAs and circRNAs could yield additional potential targets for treatment (Gao M. et al., 2022).

**FIGURE 1:**
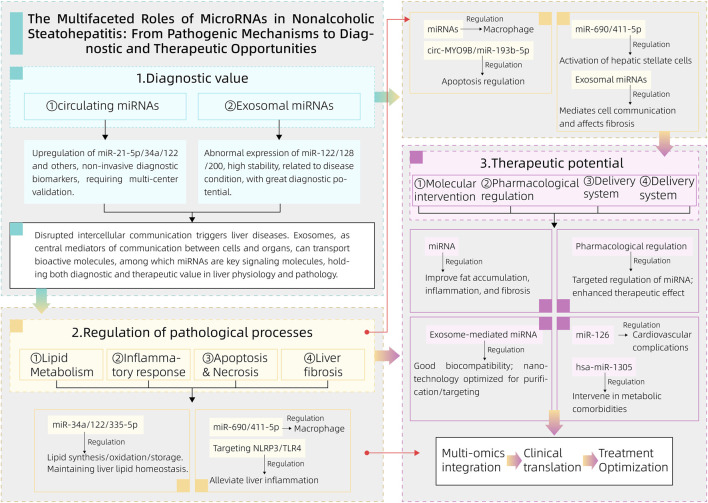
Graphical abstract summarizing the multifaceted roles of miRNAs in NASH. This schematic illustrates the three major aspects of miRNA involvement in NASH: (1) Diagnostic value, including circulating miRNAs (upregulation of miR-21-5p/miR-34a/miR-122) and exosomal miRNAs (abnormal expression of miR-122/miR-128/miR-200), the latter exhibiting high stability and correlation with disease condition. (2) Regulation of pathological processes, including lipid metabolism (miR-34a/miR-122/miR-335-5p), inflammatory response (targeting NLRP3/TLR4 and macrophage-derived miR-690/miR-411-5p), apoptosis/pyroptosis (circ-MYO9B/miR-193b-5p), and liver fibrosis (hepatic stellate cell (HSC) activation). (3) Therapeutic potential, encompassing molecular intervention, pharmacological regulation, and exosome-based delivery systems. Disrupted intercellular communication triggers liver diseases, with exosomes serving as central mediators that transport miRNAs, which are key signaling molecules. Multi-omics integration and clinical translation are highlighted as future directions.

In summary, miRNAs participate in numerous vital aspects of NASH, including pathogenicity, diagnosis, and treatment. This molecule is stable in circulation and combines its own regulatory properties, making it a good noninvasive marker and a possible future treatment. Although there are no approved clinical therapies based on miRNAs to date, many preclinical animal studies and much initial clinical proof-of-concept evidence lay a solid foundation for the future development of diagnostics and treatments for diseases associated with miRNAs. In the near future, integrating “multi-omics” with precision medicine can help deepen understanding of miRNA mechanisms and their regulatory networks, move toward clinical transition for the diagnosis of NASH at very early stages, and design individualized care (Atic et al., 2023; Hochreuter et al., 2022; Zhu et al., 2023; Mansour et al., 2025). The multifaceted roles of miRNAs in NASH are summarized in Figure 1, which provides an overview of their diagnostic value, regulation of pathological processes (lipid metabolism, inflammation, apoptosis/pyroptosis, and fibrosis), therapeutic potential, and future directions involving multi-omics integration and clinical translation.

## 2 Main text

### 2.1 Expression patterns and diagnostic value of miRNAs in NASH

#### 2.1.1 Circulating miRNA expression profiles and diagnostic accuracy

In recent years, numerous studies have conducted thorough analyses of circulating miRNA expression profiles in serum specimens from NASH patients. These studies showed that levels of particular miRNAs are much higher in NASH patients, suggesting they can be a noninvasive diagnostic biomarker. Especially, miRNAs like miR-21-5p, miR-92-5p, miR-34a, and miR-122 have been detected repeatedly across studies. Some novel miRNAs, such as miR-193a-5p, miR-128, and miR-200, have been identified by researchers as associated with NAFLD/NASH. Higher miR-193a-5p levels correlate with greater disease activity and a worse fibrosis stage, suggesting that miR-193a-5p may be a marker of worsening disease (Hendy et al., 2019; Johnson et al., 2022).

In short, alterations in circulating miRNA expression patterns both indicate the NASH disease state and provide a fundamental foundation for an early, noninvasive diagnosis. In the future, large-scale multicenter clinical validation could lead to its clinical use and may aid in the accurate diagnosis and treatment of NASH.

Direct evidence from human studies has further challenged the assumption that circulating miRNAs are predominantly derived from injured hepatocytes. In a study analyzing miRNA expression in portal vein blood, hepatic vein blood, and liver tissue samples from patients with liver disease, three miRNAs with the most pronounced expression dysregulation, miR-146a-5p, miR-26a-5p, and miR-191-5p, showed no significant differences in expression levels across different vascular beds or between blood and liver tissue (Blaya et al., 2021). This finding strongly suggests that these circulating miRNAs are not primarily of hepatic origin, directly challenging the conventional view that circulating miRNAs are merely passive release products of liver injury.

Notably, peripheral blood mononuclear cells (PBMCs) have been identified as an important extrahepatic source of specific circulating miRNAs. In patients with liver cirrhosis, the expression of miR-26 and miR-146a was significantly downregulated in PBMCs, mirroring their expression patterns in serum (Blaya et al., 2021). This consistency suggests that PBMCs, which are key components of the immune system, may serve as a major extrahepatic source of these miRNAs. This discovery provides a new perspective for understanding the immunomodulatory role of circulating miRNAs in liver cirrhosis and its complications: these miRNAs may originate from activated immune cells, participate in systemic inflammatory responses, and thereby be associated with extrahepatic organ failure (Johnson et al., 2022; Blaya et al., 2021).

#### 2.1.2 Role of exosomal miRNAs in NASH diagnosis

Exosomes are important cell-to-cell communication messengers and carry a wide range of biomolecules (Peng et al., 2021). Amidst this, miRNAs have become a focus for NASH identification due to their high stability and distinctive expression patterns. Exosomal miRNA resides in a lipid bilayer membrane, so it is not broken down into pieces by nuclease in the blood; it is more stable and has more meaning than circulating miRNA. Its expression status reflects the hepatic pathological state; it plays a key role in regulating liver inflammation, hepatic fibrosis, and metabolic deregulation (Morishita et al., 2023; Mahmoudi et al., 2022). Numerous studies have shown that exosomal miRNAs, including miR-122, miR-128, miR-200, miR-298, and miR-342, are aberrantly expressed in NASH. Moreover, the expression levels of those miRNAs in plasma or serum exosomes are closely linked to disease severity and show good diagnostic potential. High-throughput studies showed that a few stably differentially expressed miRNAs were detected in the circulation and exosomes of NASH patients. All candidate biomarkers and their diagnostic abilities are included in Table 1.

**TABLE 1:** Candidate circulating and exosomal miRNA biomarkers for the diagnosis of NASH.

miRNA name	Expression trend	Sample type	Diagnostic performance (e.g., AUC)	Pathological association	References
miR-21-5p	Upregulated	Serum	Panel AUC >0.87	Inflammation, oxidative stress	Kim et al. (2021)
miR-122	Upregulated	Serum, exosome	Correlates with disease severity	Lipid metabolism, inflammation	Atic et al. (2023); Hochreuter et al. (2022); Hwang and Yang (2021)
miR-34a	Upregulated	Serum	Potential diagnostic marker for NASH	Lipid metabolism, inflammation	Atic et al. (2023), Hendy et al. (2019)
miR-192-5p	Upregulated	Serum	Panel AUC >0.87	Distinguishes NAFL from NASH	Kim et al. (2021)
miR-128-3p	Upregulated	Liver-derived exosomes	C-statistic >0.78	Positively correlates with NASH severity	Newman et al. (2022)
miR-411-5p	Downregulated	Serum exosomes	Negatively correlates with fibrosis	Inhibits HSC activation	Wan et al. (2022)

This is a list of miRNAs that show differential expression in serum, plasma, or exosomes between patients with NASH and healthy controls or between patients with steatosis and healthy controls. Abbreviations: AUC, area under the receiver operating characteristic curve; HSC, hepatic stellate cells; NAFL, nonalcoholic fatty liver.

Exosome miRNA is highly stable and can reflect the pathological condition of the liver, so it is a strong indication for a noninvasive NASH diagnostic biomarker. Especially the specific expression changes of miRNAs like miR-122, miR-128, miR-200, miR-298, miR-342, etc., in NASH patients, combined with the regulatory mechanisms of the LPS/TLR-4/FoxO3 signaling pathway, provide novel approaches and means for clinical early diagnosis, disease staging, and monitoring of treatment efficacy, having broad application prospects (Morishita et al., 2023; Mahmoudi et al., 2022). Future studies should continue testing the clinical application of these exosomal miRNAs and explore them for the treatment of NASH.

Dysregulation of intercellular communication constitutes a pivotal mechanism in the pathogenesis and progression of numerous hepatic disorders. In recent years, extracellular vesicles, particularly exosomes, have garnered significant attention as key mediators of intercellular communication (Zhang et al., 2020). These nanoscale vesicles can transport bioactive molecules, including proteins, lipids, and nucleic acids, facilitating the exchange of information between cells and even organs (Li et al., 2021). Among these, miRNAs, a crucial class of non-coding RNAs, serve as core signaling molecules in exosome-mediated intercellular communication (Yang et al., 2018). They play a dual role in both physiological and pathological liver processes: acting as messengers of cellular status, they can serve as biomarkers for disease diagnosis; simultaneously, they function as regulators of cellular function, influencing the fate of recipient cells by modulating target gene expression, thereby emerging as potential therapeutic targets (Lee et al., 2022).

Beyond their diagnostic utility, exosomal miRNAs actively participate in intercellular communication networks that drive pathogenesis. MiRNA transfer between hepatocytes, hepatic stellate cells (HSC), and macrophages is mainly mediated by exosomes, which play a key communication role in the pathogenesis of liver diseases (Hwang and Yang, 2021). Exosomes, as vesicles of 50–150 nm, carry molecules such as miRNA and regulate gene expression in recipient cells. In non-malignant liver diseases, the number of exosomes derived from hepatocytes increases and the miRNA spectrum changes, which affects the activation of HSC and the function of macrophages, thus regulating the process of inflammation and fibrosis (Lee et al., 2022). These findings reveal the potential of intercellular miRNA metastasis as a biomarker for the diagnosis and treatment of liver diseases and provide new ideas for developing intervention strategies for non-malignant liver diseases (Lee et al., 2022; Hwang and Yang, 2021).

### 2.2 Pathological mechanisms regulated by miRNAs

#### 2.2.1 Regulation of lipid metabolism

**FIGURE 2:**
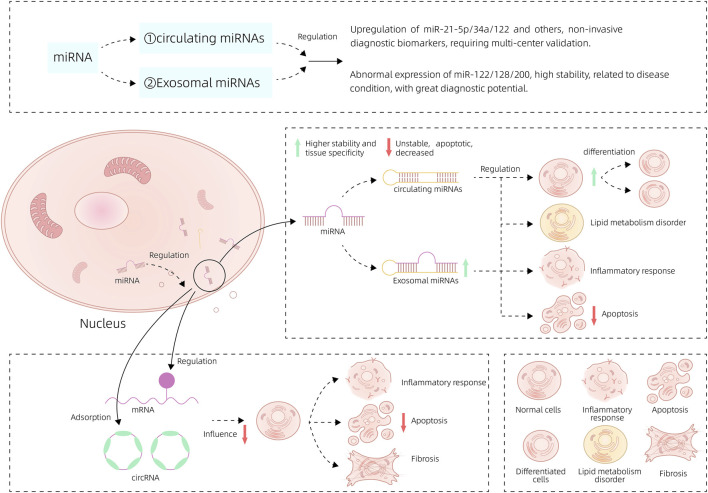
Mechanisms of miRNA-mediated regulation in NASH pathogenesis. This figure illustrates the dual diagnostic roles of circulating and exosomal miRNAs, as well as the regulatory functions of miRNAs in key pathological processes. Circulating miRNAs (e.g., miR-21-5p/miR-34a/miR-122) serve as noninvasive diagnostic biomarkers requiring multicenter validation. Exosomal miRNAs (e.g., miR-122/miR-128/miR-200) exhibit high stability and are closely associated with disease conditions. miRNAs regulate lipid metabolism disorders, inflammatory responses, apoptosis, and fibrosis. Additionally, circRNAs can adsorb miRNAs, thereby influencing the expression of target mRNAs and affecting downstream pathological processes.

MiRNAs play an important role in regulating lipid metabolism in NASH and NAFLD. miR-34a, miR-122, and miR-335-5p, specifically, can affect hepatic lipid metabolic equilibrium by regulating fatty acid biosynthesis,β-oxidation, lipid storage-related genes involved in hepatic fat accumulation regulation, and hepatocellular inflammation progression (Fang et al., 2021; Abdelgwad et al., 2023; Fan et al., 2021). The regulatory mechanisms of miRNAs in NASH pathogenesis are detailed in Figure 2, which illustrates the diagnostic roles of circulating and exosomal miRNAs, as well as their involvement in lipid metabolism disorders, inflammatory responses, apoptosis, and fibrosis. The figure also highlights how circRNAs adsorb miRNAs to influence target gene expression.

MiR-20a-5p also participates in lipid metabolism modulation. It modifies target genes such as PPARα (peroxisome proliferator-activated receptor-alpha) and CD36, which affect fatty acid oxidation and uptake and, consequently, hepatic lipid homeostasis. The cut of miR-20a-5p results in inferior fatty acid metabolism and improves hepatobiliary fat gain (Zhang H. et al., 2021; Wang et al., 2020; Lee et al., 2021).

MiRNA groups such as miR-34a, miR-122, and miR-335-5p maintain the balance of hepatic lipid metabolism by regulating genes involved in fatty acid synthesis, beta-oxidation, and lipid storage. Given that miR-335-5p is included in the list of elements associated with lipid metabolism disorders, reducing its expression increases lipid levels, leading to liver steatosis and promoting the progression of NASH. Future studies on how these miRNAs regulate the process and mechanisms could reveal entirely new molecular targets and therapeutic targets for NASH diagnosis and treatment (Mansour et al., 2025; Zheng et al., 2024; Abdelgwad et al., 2023).

#### 2.2.2 Regulation of inflammatory responses

Inflammatory responses are an important aspect of NASH pathogenesis, in which the activation of immune cells and the consequent release of inflammatory mediators jointly contribute to hepatic damage and disease progression. Many miRNAs regulate inflammation by altering Kupffer cell (liver macrophage) and other macrophage behavior, altering their inflammatory state, and thereby driving changes. The research shows that miRNAs such as miR-690, miR-193b-5p, and miR-411-5p exhibit abnormal expression and are strongly associated with NASH and animal models. Severe obesity-related NAFLD in adolescents, in which miR-411-5p was downregulated in NASH and multiple pathological features, including hepatic inflammation and fibrosis, which may regulate the hepatic inflammatory and fibrotic process (Gao H. et al., 2022; Li et al., 2024).

The NLRP3 inflammasome and TLR4 play important roles in the inflammatory response in NASH. Various studies show that miRNAs can block this pathway from being activated and releasing downstream inflammatory factors when targeted at these particular molecules, thereby preventing the liver from becoming inflamed. miR-29a improves mitochondrial function, reduces oxidative damage by targeting the MCJ gene, decreases NLRP3 inflammasome activity, eases inflammation and fibrosis in hepatic cells, and so on. Further examination found that miR-21-5p is elevated and drives inflammation and oxidative stress in NAH. Activation of PPARγ changes the PPARγ/miR-21-5p/SFRP5 pathway, lowers miR-21-5p, halts inflammatory responses, and could direct TLR4-related inflammatory signaling loops (Zhang X. et al., 2021). Endotoxins in the blood caused by gut microbiota dysbiosis stimulate TLR4 signaling, leading to hepatic inflammation. Additionally, related miRNAs like miR-122, miR-128, etc., have also been found to be close to the LPS/TLR4/FoxO3 pathway and suggest that miRNA regulation of the LPS/TLR4/FoxO3 pathway is important for alleviating liver inflammation (Samy et al., 2024).

They could serve as targets for the identification of new NASH-inflammation regulatory pathways when we develop medicines or therapeutics for these pathways. In the future, as an in-depth understanding of the exact role miRNAs play in the inflammatory signaling network and its regulation emerges, it is likely that more precise diagnoses and treatments for NASH will come about.

#### 2.2.3 Apoptosis and pyroptosis

Apoptosis and pyroptosis are key pathological mechanisms in NASH pathogenesis, directly impacting the degree of hepatocyte damage and progression levels. MiRNAs, as important epigenetic regulators, modulate the balance between cell survival and death in hepatocytes by regulating genes encoding apoptotic factors, thereby altering hepatic inflammation and scarring (Golchin et al., 2025). The amplification of hepatocyte death simultaneously causes structural damage and the release of pro-inflammatory mediators, which activate Kupffer cells and stellate cells, thereby stimulating the production of liver fibrosis. It is one of the reasons for NASH cirrhosis (Chen et al., 2024).

However, circ-MYO9B itself affects liver cell apoptosis by modulating lipid metabolism and inflammatory responses, as it induces miR-193b-5P expression. The analysis of circ-MYO9B and miR-193b-5p expression in blood and liver tissue from NASH patients showed a positive correlation between them in NASH-related cirrhosis patients (Golchin et al., 2025; Chen et al., 2024). RT-qPCR shows they do have abnormal expression, which plays an important role in disease progression. This miRNA exerts regulatory effects that not only accelerate hepatocyte apoptosis but also trigger pyroptosis, a form of inflammatory cell death. Pyroptosis, triggered by inflammasome activation, induces altered membrane permeability and pro-inflammatory cytokine release, exacerbating the hepatic inflammatory microenvironment and accelerating NASH progression (Golchin et al., 2025; Chen et al., 2024). Related lncRNAs, including MIR22HG (Chen et al., 2024), may promote autophagy and inhibit pyroptosis, fibrosis, and NASH development by modulating the miR-9-3P-IGF1 pathway via ceRNA. This study showed that lower MIR22HG expression is associated with greater pyroptosis in liver tissue from NASH patients, suggesting it may serve as a marker for detection and therapy (Chen et al., 2024). MiRNAs work together to regulate hepatitis cell death and pyroptosis through multiple targets and pathways. They act as important central points for linking problems with how we get energy, how our bodies fight off diseases, and how the liver is damaged. This shows an important regulatory layer in the pathological process of NASH.

MiRNA mechanisms regulating hepatocyte apoptosis and pyroptosis in NASH have significant research and clinical value; circ-MYO9B modulates lipid metabolism and inflammation via miR-193b-5p to affect apoptosis and thus has considerable therapeutic potential (Golchin et al., 2025). Moreover, miRNAs modulate pyroptosis pathways, thereby regulating hepatic inflammation and fibrosis, providing new molecular targets and therapeutic options for comprehensive NASH management. This will help us better understand how NASH causes cell death and explore ways to prevent it (Golchin et al., 2025; Chen et al., 2024).

#### 2.2.4 Progression of fibrosis

Liver fibrosis is a significant pathological stage in NASH progression; it primarily involves the activation of hepatic stellate cells (HSCs) and their conversion into myofibroblasts. It causes a considerable amount of collagen and extracellular matrix components to be produced, which disrupt the liver’s structure and function. MiRNAs are an important class of non-coding RNAs that regulate gene expression. miRNAs play an important role in hepatic fibrosis. According to reports, miR-690, miR-411-5p, and others are involved in regulating hepatic fibrosis by modulating the activation state of HSCs and the expression of target genes in these cells. Specifically, miR-411-5p expression is much lower in the serum exosomes of people with NASH and is reduced in liver tissues and in M2 macrophage cells within the livers of NASH mouse models. Inhibiting HSC activation directly by regulating CAMSAP1 expression slows the development of liver fibrosis. Knockdown of CAMSAP1 also inhibits HSC activation, suggesting that CAMSAP1 may be a therapeutic target for NASH (Wan et al., 2022).

The exosome-facilitated intercellular communication mechanism plays an important role in liver fibrosis pathogenesis. Like nanoscale membrane vesicles secreted by cells, exosomes can carry bioactive molecules such as miRNAs for intercellular communication. In terms of NASH-related liver fibrosis, macrophage-derived exosomal miRNAs affect the activation status of HSCs and influence the hepatic inflammatory and fibrotic processes. Research indicates that exosomal miRNAs serve as noninvasive diagnostic markers and have therapeutic potential for treating fibrosis. Studies also show overexpression of certain miRNAs, such as miR-221, in the fibrotic liver. Inhibition of miR-221 reduces the expression of miR-221 target fibrosis genes and HSCs (Markovic et al., 2020). Additionally, miR-155-deficient mice showed greatly reduced hepatic injury and fibrosis in the high-fat, high-cholesterol, high-glucose diet-induced NASH model, suggesting that miR-155 may play a role in the development of NASH-related fibrosis (Bala et al., 2021). The data suggest that miRNAs regulate HSC activation and fibrosis progression by modulating key target genes and pathways.

Altered miRNA expression is associated with different fibrosis-proliferative signaling pathways, including TGF-β, Wnt/β-catenin, and Hippo. NASH fibrosis-specific circRNA–miRNA–mRNA regulatory networks have been reported, such as the circ608–miR-222–PINK1 axis, which controls mitochondrial autophagy through HSC and then the fibrosis process (Xu et al., 2022). miR-34a-5p can also regulate GREM2 and inhibit the TGF-β pathway; finally, empagliflozin improves the progression of NASH fibrosis (Shen et al., 2023). Such multi-level miRNA-regulating networks highlight the intricate molecular mechanisms behind hepatic fibrosis, offering new directions for targeted therapeutics.

MiRNAs play a role in regulatory fibrosis-related signaling pathways and also serve as diagnostic and therapeutic targets, providing a basis for experimental studies aimed at early diagnosis and precise treatment of NASH fibrosis (Wan et al., 2022; Markovic et al., 2020; Bala et al., 2021; Xu et al., 2022; Shen et al., 2023; Tadokoro et al., 2021). Table 2 shows that a single miRNA may participate in multiple pathological processes. miR-122 is a regulator of lipidaemias and also participates in the pro-inflammatory response, making it complex. Studies on miR-690 from Kupffer cells and miR-411-5p in M2 macrophage exosomes also highlight the relevance of cellular communication in NASH development and provide new avenues to target the disease.

**TABLE 2:** Functional roles of microRNAs in the pathogenesis of NASH.

Pathological process	miRNA name	Expression trend	Target gene/Pathway	Primary function	References
Lipid metabolism	miR-34a	Upregulated	FASN and other lipid metabolism genes	Promotes fatty acid synthesis and lipid accumulation	Mansour et al. (2025); Wan et al. (2022)
	miR-122	Upregulated	FASN, β-oxidation enzymes	Regulates lipid homeostasis; influences steatosis	Zheng et al. (2024); Fang et al. (2021)
	miR-335-5p	Downregulated	ACACA, FASN	Its downregulation promotes aberrant lipid metabolism and deposition	Abdelgwad et al. (2023)
Inflammation	miR-690	Downregulated	NADK	Suppresses Kupffer cell-mediated inflammation and fibrosis	Gao et al. (2022b)
	miR-193b-5p	-	Regulated by circ-MYO9B	Modulates lipid metabolism and inflammatory response	Golchin et al. (2025)
Fibrosis	miR-411-5p	Downregulated	CAMSAP1	Inhibits hepatic stellate cell (HSC) activation	Wan et al. (2022)
	miR-155	Upregulated	-	Promotes fibrosis; its deficiency alleviates fibrosis	Bala et al. (2021)

The table lists major miRNA functions based on their role in major pathological mechanisms of NASH, such as metabolic, inflammatory, and fibrotic changes, as well as cell death. Abbreviations: ER, endoplasmic reticulum; HSC, hepatic stellate cell; LPS, lipopolysaccharide; TLR4, toll-like receptor 4.

### 2.3 MiRNA and ceRNA networks

#### 2.3.1 Construction of the circRNA–miRNA–mRNA regulatory network

With recent developments in high-throughput sequencing technology and bioinformatics applications, the circRNA-miRNA-mRNA regulatory network has been widely used to study the mechanisms underlying many diseases, including tumors, immune diseases, cardiovascular diseases, and metabolic diseases. CircRNA is a closed-loop type of non-coding RNA that can adsorb miRNA, liberate the effect of miRNA inhibition, and form a complex ceRNA network. This regulatory model has more layers than traditional gene expression regulation and also provides new insights into disease mechanisms and therapeutic strategies (Khan et al., 2021; Liang et al., 2020).

Similar findings have been made through other studies on diseases such as systemic lupus erythematosus, cardiovascular disease, and tumors, which have all shown that these circRNA-miRNA-mRNA regulatory networks play a central role in the development and progression of these diseases. For example, in systemic lupus erythematosus, we constructed the circRNA-miRNA-mRNA network for the above patients to identify many important circRNAs and miRNAs, and we suggest that these are essential for regulating autoimmunity (Khan et al., 2021). In the tumor field, we establish a ceRNA network using multi-omics data, uncovering the molecular mechanisms of various tumors and providing new molecular markers and target genes for tumor diagnosis and therapy (Zuo et al., 2021; Zhou et al., 2020).

In terms of technicalities, most researchers obtain their expression data from common repositories such as GEO and TCGA. Differential expression analysis is used to identify key circRNAs, miRNAs, and mRNAs, and then test these factors using several predicted tools (miRanda, TargetScan, miRDB) to examine interaction relationships and build a complete regulatory network. In addition, performing functional enrichment analyses such as GO and KEGG will provide explanations of biological functions and pathways, further supporting the biological significance of genes identified in the network (Gao J. et al., 2022; Chao et al., 2021).

In summary, we have identified circRNA–miRNA–mRNA regulatory networks that enable us to better understand the progression of NASH and related processes. Finding important circRNAs and the factors that control them can help determine how diseases work at a molecular level, find new ways to detect diseases and develop new treatments, and develop tests to determine whether someone has a disease and specialized ways to treat them. In the future, it might be possible to perform experimental validation using clinical samples to improve how we explain what this network does and to start using this regulatory network in NASH (Gao M. et al., 2022; Zhang J. et al., 2021).

#### 2.3.2 Key miRNAs and their target genes

NASH has a complicated mechanism. Various pathophysiological changes occur simultaneously, disrupting lipid metabolism, increasing inflammation, and promoting liver fibrosis. Over the past few years, it has been discovered that miRNAs (microRNAs) play an important regulatory role in gene expression and are involved in the pathogenesis of NASH. Research shows that miRNAs such as hsa-miR-335-5p, miR-582-5p, and miR-292a-3p play key regulatory roles in the NASH ceRNA network. These miRNAs affect NASH progression through lipid metabolism, inflammation, and fibrosis.

In addition to being at the center of the NASH ceRNA network, miRNAs also directly target and regulate key genes in lipid metabolism, inflammation, and fibrosis-related signaling pathways, thereby affecting the onset and progression of disease. These miRNAs will lead to changes in hepatocyte function, influence liver fibrosis in HSCs and hepatic stellate cells to some degree, and be associated with lipid storage and the production of inflammatory factors, making them a target for NASH diagnosis and treatment. Regulatory strategies targeting these miRNAs in the future offer new approaches and methods for precision treatment of NASH (Abdelgwad et al., 2023; Xiang et al., 2022; Huang et al., 2024). The key miRNAs identified within ceRNA networks and their regulatory roles are summarized in Table 3.

**TABLE 3:** Key miRNAs within ceRNA networks in NASH.

Key miRNA	Role in network	Regulated biological process	Target gene/Interacting molecule	References
hsa-miR-335-5p	Upstream regulator of nine hub lipid metabolism genes	Lipid metabolism	Multiple lipogenesis genes	Cha et al. (2021)
miR-582-5p/-3p	Associated with NAFL-to-NASH progression; modulates HSC function	Liver fibrosis, gut microbiota	TMBIM1	Zuo et al. (2021); Zhou et al. (2020)
miR-292a-3p	Forms a ceRNA network with lncRNAs involved in the steatosis-to-NASH transition	Lipid metabolism, inflammation, apoptosis	Multiple NASH-related genes	Zuo et al. (2021)

This table lists miRNAs identified as key nodes in ceRNA regulatory networks (such as circRNAs or lncRNAs) by multi-omics studies of NASH. Abbreviations: ceRNA, competitive endogenous RNA; circRNA, circular RNA; HSC, hepatic stellate cell; lncRNA, long non-coding RNA.

### 2.4 Research progress on miRNAs as therapeutic targets for NASH

#### 2.4.1 MiRNA inhibitors and mimetics

MiRNA inhibitors and mimetics have great potential as emerging molecular interventions in NASH therapeutic research. miRNAs affect fatty liver metabolism, the inflammatory response, and scarring by altering the expression of gene targets; thus, miRNAs are a promising target for regulating the pathologic progression of NASH. Current studies focus on specific miRNA inhibitors and mimics to restore normal miRNA expression, restore liver function, and reduce disease burden.

In summary, miRNA inhibitors and mimics showed varied effects in NASH animal models, reducing hepatic lipid storage, controlling inflammation, and hindering fibrosis progression. There is great potential for new clinical methods for NASH in terms of future developments of miR-34a inhibitors combined with specific transporters and miR-690 mimics with transport. At the same time, due to miRNAs’ multiple targets and complex regulatory network, exploring miRNA mechanisms of action and conducting comprehensive safety evaluations remain very important for the clinical application of miRNAs (Zhu et al., 2023; Gao H. et al., 2022).

#### 2.4.2 Pharmacological regulation of miRNA expression

Pharmacological modulation of miRNA expression is an ongoing research area for NASH treatment. Studies have shown that controlling specific miRNA expression can stop hepatic inflammation and fibrosis.

Moreover, combination therapies aiming for miRNA regulatory pathways are also showing promise. To identify drugs that can be combined for better treatment, we could use bioinformatics and systems biology to analyze the miRNA regulatory network and predict which drugs can be combined to improve treatment. Consider naringenin and puerarin, for example: they are specific natural products capable of modulating several miRNA-related signaling pathways to help medicine comprehensively control the liver’s abnormal lipid metabolism and irregular inflammatory response. Using this multi-target combination therapy could help overcome some of the problems with using a single medicine to treat NASH (Li et al., 2023; Yu et al., 2023). In future research incorporating miRNA expression with drug mechanisms, theory can support individualized NASH treatments, and miRNA-centered combination therapies can advance in clinics.

MiRNA pathway-based drug combination prediction shows that combination therapy has great potential and can provide new strategic directions for the precision treatment of NASH. These outcomes both improve our understanding of miRNAs’ role in NASH development and set the stage for the development of more effective miRNA-focused medicines (Abdelgwad et al., 2023; Sun et al., 2023).

#### 2.4.3 Exosome-mediated miRNA delivery systems

Exosomes are very important carriers of cell-to-cell communication. They contain many biologically active compounds, such as proteins, mRNA, and miRNA, that are involved in many healthy and unhealthy situations. In recent years, using exosomes as a promising carrier system for disease therapy has emerged as a new approach with good biocompatibility, low immunogenicity, and target specificity; exosomes are also effective carriers for nucleic acid drugs (Balaraman et al., 2025). Compared with standard liposomal or viral carriers, exosomes provide better protection for miRNA against degradation during *in vitro* processes, thereby enhancing its stability and bioefficacy. In addition, they can be delivered to some cells by modifying surface proteins, thereby improving targeted precision and efficiency (Del Pozo-Acebo et al., 2021; Li et al., 2022).

Exosomes’ main mission is to carry target miRNA mimics or inhibitors, so miRNAs will not lose their functions afterward. The common methods of loading exosomes are electroporation, transfection, and cell processing, and when miRNA is loaded onto an exosome, it is greatly increased. One approach is to manipulate mesenchymal stem cells to overexpress specific miRNAs, enriching these miRNAs in secreted exosomes to produce natural carriers, as reported by Vakhshiteh et al. (2020), Sohrabi et al. (2022), and Han et al. (2021). Altering their surface by attaching tumor-related ligands or antibodies to exosomes can enable targeted delivery, thereby making miRNA delivery more effective (Li et al., 2022; Han et al., 2021).

With respect to researching and treating NASH and liver fibrosis, what has been made available by this exosome-delivered miRNA is considerable; studies indicate that exosomes can transport active miRNAs to hepatocytes, so they may regulate inflammation, fat metabolism, and fibrosis events, thereby reducing the activity of the disease (Qi and Lai, 2022; Chen et al., 2025). Consider exosomal miR-26a delivery to hepatocytes as an example. It can inhibit the progression of hepatocellular carcinoma and exert antitumor effects (Mahati et al., 2021). It has delivered exosome-based miRNAs to various diseases, such as cancer and cardiac disease, and achieved positive results (Balaraman et al., 2025; Liu et al., 2022).

However, exosome-transported miRNA delivery still faces some tough problems, such as significant challenges in clearing large numbers of exosomes, improved loading capacity, improved tissue distribution, and targeting specific goals, as well as consideration of immune system concerns and potential off-target effects (Huang et al., 2024; Han et al., 2021). Thus, many scientists have worked to utilize nanotechnology techniques in modifying exosomes through a combination of genetic engineering and chemical modification techniques to develop exosome delivery systems with responsiveness and targeted delivery (Wang et al., 2024; Yin et al., 2024), which is expected to realize efficient, safe, and accurate miRNA delivery.

**FIGURE 3:**
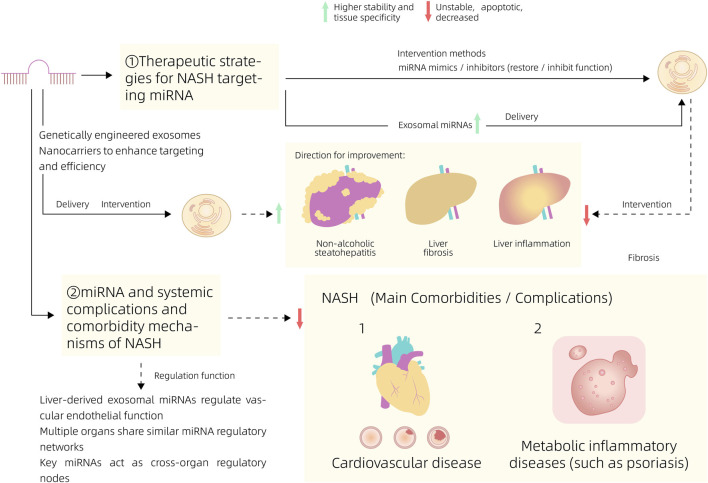
Therapeutic strategies and systemic complications of miRNAs in NASH. Left panel: Major therapeutic intervention strategies targeting miRNAs, including miRNA mimics/inhibitors, exosomal miRNA delivery, genetically engineered exosomes, and nanocarriers to enhance targeting and efficiency. Right panel: Systemic complications and comorbidities associated with NASH. Liver-derived exosomal miRNAs regulate vascular endothelial function, contributing to cardiovascular disease. Multiple organs share similar miRNA regulatory networks, with key miRNAs acting as cross-organ regulatory nodes, linking NASH with metabolic inflammatory diseases such as psoriasis.

In conclusion, exosomes have significant potential as miRNA carriers, increasing miRNA targeting and therapeutic effects. Improvements in the preparation, loading, and targeting of exosomes may improve the application of miRNA gene therapy to treat liver diseases like NASH. The experimental therapeutic strategies targeting miRNAs in NASH, including inhibitors, mimics, and exosome-mediated delivery, are summarized in Table 4. The major therapeutic intervention strategies targeting miRNAs are illustrated in the left panel of Figure 3, while the systemic complications and comorbidities associated with NASH are summarized in the right panel.

**TABLE 4:** Experimental therapeutic strategies targeting microRNAs in NASH.

Therapeutic strategy	Target miRNA	Intervention	Model	Key findings	References
miRNA inhibitor	miR-34a	Antagonist	Animal model	Ameliorates hepatic steatosis, inflammation, and fibrosis; improves liver enzymes	Zhu et al. (2023)
miRNA mimic	miR-690	Mimic	Animal model	Suppresses HSC activation; attenuates fibrosis and steatosis	Gao et al. (2022b)
Pharmaceutical modulation	Multiple (e.g., miR-21a-5p)	Long-acting pirfenidone	Experimental study	Downregulates pro-fibrotic/inflammatory miRNAs, suppressing inflammation and fibrosis	Li et al. (2023)
Exosome-mediated delivery	miR-411-5p	M2 Macrophage-derived exosomes	*In vitro*/Animal model	Inhibits HSC activation by targeting CAMSAP1	Wan et al. (2022)

Experimental studies exploring therapeutic approaches involving microRNAs (miRNAs) in NASH, including inhibitors, mimics, and pharmacological modifiers, in preclinical studies. Abbreviations: ALT, alanine aminotransferase; AST, aspartate aminotransferase; HSC, hepatic stellate cell.

#### 2.4.4 Clinical translation of miRNA-based strategies for NASH

Much evidence shows that NAFLD is closely related to the change of miRNA expression pattern in the liver in the early, middle, and late stages of the disease (Hochreuter et al., 2022). Specific miRNA species, such as miR-34a, miR-122, and miR-21, are directly involved in the progression of steatosis, NASH, and liver fibrosis. These miRNAs both play a role in liver cells and serve as intercellular messengers, transmitting signals between liver cells and other liver cells (such as hepatic stellate cells and Kupffer cells), and even serve as inter-organ messengers in circulation, connecting the liver with extrahepatic metabolic tissues such as adipose tissue and the cardiovascular system (Hochreuter et al., 2022). In addition, recent research identified inflammatory and NET-related differentially expressed genes (INRDEGs), including TNF, IL-6, CCL5, and SPP1, through bioinformatics analysis. These genes were verified in a NASH mouse model, and their serum levels increased significantly (Zhang et al., 2025). These findings reveal the complex molecular regulatory network of NAFLD/NASH.

In view of the invasiveness and limitations of liver biopsy, developing reliable serum biomarkers is the key to clinical transformation. At present, miR-34a, miR-122, and miR-192 are considered the best candidate biomarkers for the diagnosis and staging of NAFLD (Hochreuter et al., 2022). These miRNA changes can reflect liver injury and disease progression. In addition, a diagnostic model based on four serum biomarkers, TNF, IL-6, CCL5, and SPP1, was established in one study, which showed high accuracy in distinguishing NASH patients (Hochreuter et al., 2022; Zhang et al., 2025). More importantly, these markers can also achieve risk stratification: TNF, SPP1, and CCL5 mark high-risk patients, while IL-6 dominates the low-risk subgroup, and two NAFLD subtypes with different immune characteristics are identified. This provides a noninvasive tool for precision medicine (Zhang et al., 2025).

It is often difficult for a single biomarker to fully reflect the complexity of NASH. Studies have shown that the accuracy of diagnosis and risk stratification can be significantly improved by adopting a multi-marker combination strategy, such as jointly detecting miR-34a, miR-122, and miR-192, or using a biomarker panel composed of TNF, IL-6, CCL5, and SPP1 (Hochreuter et al., 2022; Zhang et al., 2025). This multi-marker method is helpful for noninvasive diagnosis and can realize the classification of disease subtypes, thus guiding personalized treatment choices. For example, for high-risk subgroups (high expression of TNF, SPP1, and CCL5), anti-inflammatory or anti-fibrosis therapy can be given priority. The low-risk subgroup (high IL-6 expression) may need different intervention strategies (Zhang et al., 2025). This accurate stratification based on biomarkers is a key step to realize NASH personalized medical care.

The clinical transformation based on miRNA strategy in NASH is in a critical period full of opportunities and challenges. Circulating miRNAs (such as miR-34a, miR-122, and miR-192) and inflammation-related proteins (such as TNF, IL-6, CCL5, and SPP1) have been identified as potential biomarkers for noninvasive diagnosis and risk stratification, and the multi-marker combination strategy has laid a solid foundation for precision medicine.

### 2.5 MiRNA and NASH-related complications and systemic effects

#### 2.5.1 Cardiovascular disease risk

NASH patients tend to have a very high risk of cardiovascular disease, which has greatly excited the world’s academic community studying the liver and cross-organ communications with the cardiovascular system. Research finds that liver-secreted exosomal miRNAs have a significant influence on regulating vascular endothelial function in NASH patients with abnormal miRNA expression. NASH mouse studies show that the new exosomal microRNA-126 promotes inflammation and endothelial dysfunction via the AhR–NLRP3 pathway (Chen et al., 2025).

This finding elucidates the pathogenesis of NASH and cardiovascular disease, and it shows how the liver controls the cardiovascular system via miRNA secretion, revealing a cross-organ communication pathway. This means that distant organs can be affected by what happens to the liver, so if we target miRNA expression or its post-transcriptional effects, we might be able to fix cardiovascular problems in NASH patients. We know that miRNAs can regulate gene expression and play a substantial role in nonalcoholic fatty liver disease (NAFLD) progression and the risk of cardiovascular complications. There are many genome-wide association studies (GWASs) and useful functional animal experiments demonstrating that miRNAs can serve as disease biomarkers, but their clinical application requires further validation and optimization (Jonas and Schürmann, 2020).

MiRNAs influence NASH cardiovascular disease, which gives grounds for creating new medical tests and treatments. Precisely regulating miRNAs, especially targeting extracellular miR-126 and its control over the AhR/NLRP3 signaling pathway, might be an important method of preventing and reducing heart disease in people with NASH. At the same time, this mechanism deepens our understanding of the complex interactions between the liver and cardiovascular systems, bringing new ideas for the treatment of coordinated disorders across multiple organs (Chen et al., 2025; Jonas and Schürmann, 2020).

#### 2.5.2 Mechanisms of comorbidity with other metabolic diseases

As the severe progressive stage of NAFLD, NASH exhibits intricate comorbidity mechanisms with multiple metabolic disorders. Bioinformatics analyses reveal overlapping gene expression and miRNA regulatory networks between NASH and immune-inflammatory diseases such as psoriasis. This overlap between co-expressed genes and miRNA networks suggests common pathological mechanisms across diseases, especially among those that share similarities in the way their inflammatory responses and metabolic imbalances occur. Studies have shown that using weighted gene co-expression network analysis (WGCNA) on high-throughput data and screening for differentially expressed miRNAs can identify more than one important miRNA regulating NASH and other metabolic inflammatory diseases. It showed that miRNAs act as a link that transmits regulatory signals among different pathologies (Golchin et al., 2025).

Among them, hsa-miR-1305 is one of the co-expressed miRNAs involved in regulating multiple inflammation- and metabolism-related pathways. It plays an essential regulatory role in key NASH pathophysiological processes, such as fatty acid metabolism, insulin resistance, and cell apoptosis, indicating its potential as both a biomarker of disease progression and a therapeutic target.

The complexity of the miRNA regulatory network is reflected in its extensive impact across various cell types, including hepatocytes, immune cells, and adipocytes. Cells have some interactions that are regulated by miRNA signaling. Take, for example, the many miRNAs in people with NASH that not only exhibit aberrant expression inside the liver but also appear where they are not supposed to be, thanks to being transported on exosomes in the bloodstream like passengers on tiny floating pods. This is how information is passed between the liver and other parts of the body. Consequently, this system crosstalk underlies the high comorbidity between NASH and other system-wide metabolic disorders like psoriasis, diabetes, and obesity. This means that there is some give and take between the inflammation throughout a patient’s body and problems with how different organs use energy (Hochreuter et al., 2022; Qi and Lai, 2022).

NASH’s co-expression and functional overlap with other metabolic diseases within miRNA regulatory networks provide new insights into shared disease processes. In particular, miRNA hsa-miR-1305, like other key regulators of inflammation and metabolism, can be considered a diagnostic and therapeutic biomarker. Future research could focus on a more detailed characterization of the target genes and signaling pathways of these miRNAs in other types of metabolic diseases and on cross-disease regulation. Interventions that focus on co-morbid mechanisms could further improve multi-target accuracy (Abdelgwad et al., 2023; Jiang et al., 2023). The roles of miRNAs in systemic complications and comorbidities associated with NASH are summarized in Table 5.

**TABLE 5:** Roles of microRNAs in systemic complications and comorbidities of NASH.

Related disease/Complication	Key miRNA	Mechanism of action	Pathophysiological impact	References
Cardiovascular disease	Novel miRNA-126	Exosome-mediated transfer; targets the AhR–NLRP3 pathway	Promotes vascular endothelial inflammation and dysfunction	Chen et al. (2025)
Metabolic comorbidities (e.g., psoriasis)	hsa-miR-1305	Co-expressed miRNA; regulates inflammatory and metabolic pathways	Links NASH with other metabolic inflammatory diseases	Mansour et al. (2025)

The table demonstrates how certain miRNAs, particularly those found in exosomes, affect the body as a whole. As such, there can be problems throughout the body, such as problems with the heart; NASH does not only affect liver cells. Abbreviations: AhR, aryl hydrocarbon receptor; NLRP3, NLR family pyrin domain containing 3.

### 2.6 Future research directions and clinical application prospects of miRNAs in NASH

#### 2.6.1 Multi-omics integration and miRNA functional validation

Multi-omics integration analysis is an important method for further clarifying the role of miRNAs in the development of complex diseases such as NASH. By integrating transcriptomic, proteomic, and metabolomic data to build more comprehensive microRNA regulatory networks, it would be possible to uncover and explore how microRNAs operate and interact synergistically at the molecular level. Therefore, such analysis contributes to disease onset and progression. Existing research uses hybrid analysis pipelines such as DLRAPom, which combine optimized machine learning models (e.g., Improved XGBoost) with traditional bioinformatics methods to successfully integrate multi-omics data. We have also targeted some of those key lncRNA–miRNA–mRNA regulatory axes, increasing the number of potential tools and candidate targets. This method works well on diseases like gestational diabetes, and it might work better when learning about miRNA regulatory networks for complicated diseases, as described by Shen et al. (2022). Moreover, multi-omics integration is widely used in oncology, generating miRNA–mRNA interaction networks that play crucial roles in regulating tumor development and shaping the immune microenvironment. Consider clear cell renal cell carcinoma as an example: a miRNA scoring system based on multi-omics data can predict patient survival and verify the tumorigenicity of miRNA-130a-3p by targeting SPOCK1 (Song et al., 2025).

Understanding of NASH has been improved with the fusion of omics data, which gives us an overall picture of how miRNA regulates multiple diseases, especially lipid metabolism and fibrosis inflammation. For example, constructing upstream-downstream regulatory networks of mRNA expression, miRNA expression, and metabolite changes enables the identification of key miRNAs and their target genes, offers reasoning pathways to these shifts in hepatic inflammatory fibrosis, and provides theories on how to identify and cure these conditions much earlier. Multi-omics data can help identify new biomarkers and enable the full use of machine learning and other predictive technologies to improve model accuracy and advance personalized precision medicine (Dong et al., 2025; D’Antona et al., 2024).

In terms of functional tests, cell and animal model tools cannot yet be done away with. By overexpressing suppressors *in vitro* or in cells, together with transcriptomic and proteomic analyses, it is possible to identify the miRNAs controlling downstream signaling cascades and how they affect metabolism, the inflammatory response, and apoptosis. Gene-knockout or transgenic animals, particularly mice, can be used to study the specific functions and mechanisms of important miRNAs in the *in vivo* process of NASH. This *in vivo* and *in vitro* validation does a good job of showing that the miRNAs that multi-omics identified have real-world meaning, which helps us build confidence to use them later in the clinic (Yang et al., 2021; Jousma et al., 2022).

Multi-omics integrated analysis, including transcriptomics, proteomics, and metabolomics, and functional validation in cell and animal models have become the mainstay of present miRNA research. They provide a systematic view of the miRNA regulatory network and offer a more in-depth understanding of how miRNAs impact the pathogenesis of NASH. They also provide us with stronger scientific grounding and experimental validation to pursue miRNA as a new diagnostic biomarker and therapeutic. It is this layer-by-layer, multi-perspective research approach that can significantly improve the accuracy of diagnosis and targeted treatment.

#### 2.6.2 Standardization and clinical translation of miRNA biomarkers

MiRNAs as biomarkers for disease diagnosis and prediction have attracted significant attention due to their specific features, stability, and nondestructive, noninvasive testing characteristics. Particularly with liver diseases such as NASH, there is much potential for miRNAs in medicine. However, many problems arise in the clinical translation of miRNA markers; the lack of standardized detection criteria is a key hindrance. Inconsistent sample collection, processing, storage, and miRNA separation/detection methods across studies make it difficult to replicate and compare research outcomes, seriously impeding the clinical application of miRNA markers (Sopić et al., 2024). Consequently, establishing standardized miRNA detection protocols has become an urgent priority. This involves controlling temporal and environmental conditions when collecting samples to ensure the stability of miRNAs, choosing appropriate internal reference genes, standardizing detection technologies (adopting consistent protocols for qRT-PCR, microarrays, high-throughput sequencing, etc.), and harmonizing data analysis methods. These measures are very effective at improving miRNA detection specificity and sensitivity, reducing technical error, and ensuring that data across labs are comparable (Precazzini et al., 2021; Katayama et al., 2025; Charidemou et al., 2025; Felekkis and Papaneophytou, 2024; Tao et al., 2025).

Additionally, it is necessary to conduct large-scale, multicenter medical research to determine whether miRNAs can serve as useful markers for diagnosing and predicting NASH. We should conduct trials across different populations, disease levels, and clinic environments to verify the universality and consistency of miRNA markers. Take, for example, miR-122, miR-34a, and miR-21, which are highly relevant to NASH and whose expression levels are detected in serum and exosomes. Studies have shown that they correlate with the severity of the disease, but multicenter, wide-ranging studies can still be difficult to find (Kim et al., 2021; Atic et al., 2023). Multicenter clinical validation both shows that miRNA biomarkers can accurately diagnose and predict prognosis and makes them easy to incorporate into medical guidelines, which are very useful for early diagnosis and personalized treatment of NASH (Charidemou et al., 2025; Felekkis and Papaneophytou, 2024).

Ring trials are a major cross-lab technical validation tool for handling technical and biological miRNA variability. They push for standardization of testing procedures, identify and address lab differences, and improve the relationship between miRNA levels and health outcomes, so as to speed up the use of miRNA markers in medicine (Sopić et al., 2024). Currently, few ring trials are conducted for miRNA biomarker studies, and more investment in these types of validation is needed.

Whether miRNA can go from a biomarker of interest to a NASH marker in the clinic will depend on having widely validated, standardized methods for detection. By integrating emerging multi-omics technologies and machine learning or other advanced methodologies, it is hoped that the future will develop more accurate and reliable miRNA diagnostic and prognostic models and advance miRNA-based clinical applications in NASH and other diseases (Tao et al., 2025; El-Daly et al., 2023).

Exosomes secreted by hepatocytes in the NAFLD state, along with the miRNAs they carry, play a pivotal role in intercellular communication within the liver and in disease progression. Research indicates that exosomes secreted by human hepatocellular carcinoma cells treated with palmitate exhibit significantly altered miRNA profiles, capable of activating hepatic stellate cells and promoting fibrosis-related gene expression. This suggests that lipid-toxic hepatocytes directly drive progression from simple steatosis to NASH via exosomal miRNAs (Precazzini et al., 2021). Furthermore, miR-411-5p from M2 macrophage-derived exosomes inhibits hepatic stellate cell activation by targeting CAMSAP1, while its downregulation in NASH models indicates that diminished protective communication promotes fibrosis (Katayama et al., 2025). Collectively, these findings confirm that exosomal miRNA-mediated “dialogue” within the hepatic microenvironment (hepatocytes, macrophages, stellate cells) constitutes a critical mechanism regulating the transition from NAFLD to NASH and fibrosis (Charidemou et al., 2025; Felekkis and Papaneophytou, 2024).

#### 2.6.3 Safety and efficacy assessment of miRNA-targeted therapy

MiRNA-targeted therapy serves as a newly emerged therapeutic strategy for NASH. Thus, the safety and efficacy of miRNA-targeted therapy must be assessed to support its further development. First, improving miRNA delivery systems is necessary to curtail off-target effects and immune responses. The compact size of miRNA molecules and their susceptibility to nuclease degradation lead to poor stability and non-specific distribution in the body, so delivery must address these issues (Tao et al., 2025; El-Daly et al., 2023; Lee et al., 2017; Yang J. et al., 2025). Currently, delivering miRNA using nanocarriers, liposomes, exosomes, and viral vectors enhances miRNA targeting and stability while reducing immunogenicity. Consider induced pluripotent stem cell (iPSC)-derived exosomes as an example: they can be used as miRNA carriers and have shown effective treatment with no noticeable systemic toxicity in SCI models, indicating they are safe to use as miRNA delivery tools (Abbas et al., 2024). Meanwhile, studies targeting miRNA therapy for CV diseases show the need to improve the delivery system to increase tissue specificity and avoid side effects (Saenz-Pipaon and Dichek, 2022; Dui et al., 2025). Within the NASH field, liver-specific delivery of miRNA modulators is potentially beneficial for liver-based control of inflammation and lipid metabolism, as specific signaling of lipids and related fractional synthesis rates (FSRs) and systemic side effects are reduced. As a vital branch of precision medicine, miRNA therapy demonstrates immense potential due to its capacity to simultaneously regulate multiple target genes, yet this also introduces complexity and challenges. Among these, miR-33 serves as a widely studied exemplar. Located within the intron of the SREBP gene, a key transcription factor in cholesterol metabolism, its function extends far beyond regulating cholesterol homeostasis (Rayner et al., 2010; Marquart et al., 2010). Existing research indicates that miR-33 plays a central role in diverse physiological and pathological processes, including lipid metabolism, cell cycle regulation, inflammation, autophagy, and tissue fibrosis (Cirera-Salinas et al., 2012; Fernández-Tussy et al., 2024; Price et al., 2019a; Price et al., 2021a). This extensive multi-target effect profile positions it as a potential therapeutic target for various diseases, such as atherosclerosis, metabolic liver disease, and renal disorders (Fernández-Tussy et al., 2024; Price et al., 2019a; Price et al., 2021a; Price et al., 2019b; Yang L. et al., 2025). However, miR-33 exhibits significant functional variation across different tissues (e.g., liver, macrophages, kidney, and brain), potentially yielding contradictory effects (Li et al., 2019; Price et al., 2021b).

Recent tissue-specific knockout studies have begun to resolve these contradictions and reveal the therapeutic potential of targeting miR-33 in the liver. Based on existing evidence, hepatic miR-33 plays a key role in promoting the malignant progression from NAFLD to NASH and HCC. Hepatocyte-specific deletion of miR-33 significantly alleviates the pathological process of NAFLD and inhibits HCC development by reducing lipid synthesis, promoting fatty acid oxidation, improving mitochondrial function, reducing oxidative stress, and suppressing the YAP/TAZ oncogenic pathway (Fernández-Tussy et al., 2024). These findings clearly indicate that inhibiting liver miR-33 is a potential and effective therapeutic strategy with great clinical promise in anti-obesity-associated HCC (Cirera-Salinas et al., 2012). Future research should further explore the safety and efficacy of targeting miR-33 to facilitate clinical translation. Particular attention should be paid to its dynamic expression changes during different disease stages and its synergistic regulatory network with other non-coding RNAs, improving targeting efficiency and tissue specificity by combining nano-delivery systems, and evaluating the long-term impact on lipid metabolism homeostasis and the immune microenvironment.

Notably, the function of miR-33 is highly complex. Although global miR-33 knockout mice exhibit reduced atherosclerotic plaques, they also display metabolic disorders such as obesity and insulin resistance. This “metabolic paradox” has driven researchers to explore the tissue-specific functions of miR-33 using tissue-specific knockout models, such as mice with liver-specific or Abca1-binding site mutations, which allow precise dissection of the local mechanisms of miR-33 (Marquart et al., 2010; Cirera-Salinas et al., 2012; Fernández-Tussy et al., 2024). Understanding the specific effects of miR-33 knockout both helps clarify its multifaceted roles in cardiovascular and metabolic diseases and provides an important basis for developing targeted miRNA therapies. Therefore, a thorough understanding of miR-33 therapy’s multi-target effects and tissue specificity is crucial for developing safe and effective therapeutic strategies.

Second, the duration of evaluation or the potential side effects is the second major problem in translating miRNA therapies. The complex pathophysiological process of NASH involves multiple mechanisms, including hepatocyte apoptosis, inflammatory responses, lipid metabolism disturbances, and fibrosis, so it demands multi-target regulation when targeting miRNAs. In existing studies, some miRNAs, such as miR-193b-5p, have shown promise in NASH models by regulating lipid synthesis, inflammation, and apoptosis (Golchin et al., 2025). However, miRNAs have multiple targets and multiple *in vivo* effects, which may lead to off-target effects and unexpected immune responses. Safety monitoring of miRNA therapies in preclinical and clinical research includes histopathological examination, assessment of inflammatory markers, and monitoring of immune responses. A metabolic intervention study of NASH patients, for example, which used a combination of noninvasive indicators and liver histopathology to assess the safety and efficacy of the treatment, is a significant resource for clinical application in miRNA-related therapy (Amin et al., 2022). More importantly, we can learn about safety information and learning experiences from miRNA therapies in oncology, cardiovascular diseases, and autoimmune diseases. Dosage, timing of administration, and delivery method of miRNA must all be controlled to ensure effectiveness while also being tolerated (Fortunato and Iorio, 2020; Pascual-García et al., 2025).

**FIGURE 4:**
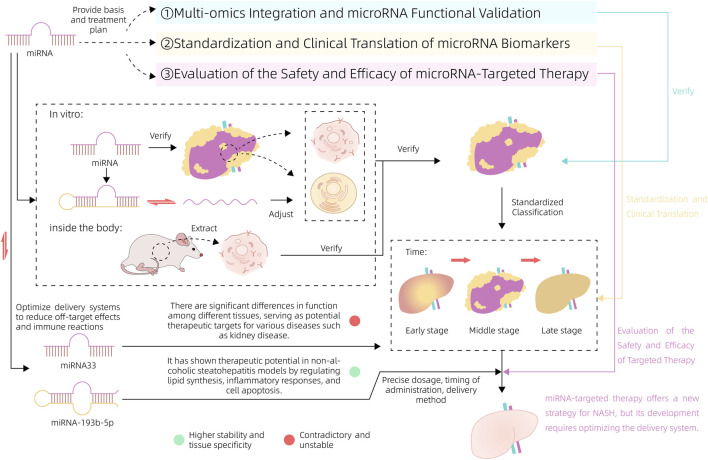
Future research directions for miRNA-based NASH diagnosis and therapy. This figure outlines three priority areas for future investigation: (1) Multi-omics integration and miRNA functional validation, requiring *in vitro* and *in vivo* verification, standardized classification, and optimization of delivery systems to reduce off-target effects and immune reactions. (2) Standardization and clinical translation of miRNA biomarkers, addressing the need for consistent detection protocols across early, middle, and late stages of disease. (3) Evaluation of the safety and efficacy of miRNA-targeted therapy, with emphasis on precise dosage, timing of administration, and delivery methods. Notable examples include miR-33, which exhibits tissue-specific functional differences and shows therapeutic potential in NASH models by regulating lipid synthesis, inflammation, and apoptosis, and miR-193b-5p, which requires higher stability and tissue specificity for clinical application. miRNA-targeted therapy offers a new strategy for NASH, but its development requires optimizing the delivery system.

Lastly, the clinical application of miRNA-targeted therapies also relies on interdisciplinary collaboration. With advances in bioinformatics, chemistry, and clinical medicine, there will be effective, safer liver-specific drug delivery systems for targeted, miRNA-specific effects. We can scientifically test the sustained effect and any potential toxicity to build good preclinical animal models and a long-term follow-up system. Advancements in nanotech, genotech, and exosome bioengineering may enable miRNA-targeted therapy to be even safer and more effective and would help translate these into practical applications for diagnosing and treating NASH patients, enabling doctors to provide truly customized care (Hochreuter et al., 2022; Stoica et al., 2025). The three priority areas for future research on miRNA-based NASH diagnosis and therapy are outlined in Figure 4: multi-omics integration and functional validation, standardization and clinical translation of biomarkers, and safety/efficacy evaluation of targeted therapy. Notably, miRNAs such as miR-33 and miR-193b-5p exemplify the therapeutic potential and challenges that require optimized delivery systems, precise dosing, and improved tissue specificity for clinical application.

## 3 Discussion

In recent years, more and more researchers have recognized that the multi-level regulation of miRNAs in NASH represents a new research direction. As crucial molecules participating in important pathological processes, such as lipid metabolism, inflammation, immune response, apoptosis, and fibrosis, miRNAs improve understanding of the complex pathological network underlying NASH and pave the way for early diagnosis and accurate therapy. Research indicates that miRNAs exert comprehensive regulatory control over various NASH pathways, affecting many targets. This sort of multi-target, multi-pathway regulation makes these possible as well as therapeutic ones. However, differences in miRNA expression and function across studies emphasize the need for careful interpretation of miRNA functional impacts. We need to strike a balance among different forms of research; we must not make things too simple or one-sided.

Circulating miRNA and exosomal miRNA are both forms of circulating miRNA. As noninvasive markers, they have greater sensitivity and specificity and offer clear advantages for early diagnosis and pathological typing of NASH. This is great progress; it provides a few handy tools for doctors to diagnose the disease, reducing the need for liver biopsies and making people more willing to accept the diagnosis. At the same time, it requires a large amount of data and is limited by a lack of common detection criteria and large-scale, multicenter validation. Thus, further studies will need to emphasize standardization in their detection, sample processing, and data interpretation techniques to enable the translation of miRNA biomarkers into a clinical setting.

In terms of therapeutic strategies, new uses for the miRNA mimic, an inhibitor, and an exosome may present new opportunities for precision therapy in NASH by changing specific miRNA expressions and suppressing abnormal pathologies with inflammation and fibrosis through various methods. They show some promising therapy. However, most current research is limited to animal studies and *in vitro* experiments, and clinical safety and efficacy have never been confirmed. Multiple, varied miRNAs with distinct regulatory networks, along with the risk of off-target effects, make more efficient, precise miRNA delivery systems imperative. At the same time, we need to consider the inter-individual differences in genetics and phenotype if we want it to be truly personal.

## 4 Conclusion

All in all, miRNAs have tremendous potential for NASH and will definitely be considered in the pathogenesis of NASH, the development of diagnostic biomarkers, and the design of new treatments. In subsequent work, we should focus more on systematic functional examination, combine with multiple omics data to deepen understanding of mechanisms, develop standardized clinical tests, reinforce multicenter clinical trial validation, and encourage the integration of miRNA and other technologies. This way, we can realize the full potential of miRNA translational applications in NASH diagnosis and treatment, helping improve how diseases are managed and making life better for people who are sick. As seasoned medical experts, we believe that in the future, miRNAs will advance from theory to practice, becoming an important part of personalized medicine for NASH.
